# Calcium Phosphate (CaP) Composite Nanostructures on Polycaprolactone (PCL): Synergistic Effects on Antibacterial Activity and Osteoblast Behavior

**DOI:** 10.3390/polym17020200

**Published:** 2025-01-14

**Authors:** Suvd Erdene Ganbaatar, Hee-Kyeong Kim, Nae-Un Kang, Eun Chae Kim, Hye Jin U, Young-Sam Cho, Hyun-Ha Park

**Affiliations:** 1 Department of Mechanical Engineering, College of Engineering, Wonkwang University, 460 Iksandae-ro, Iksan 54538, Jeonbuk, Republic of Korea; suwdka06@gmail.com (S.E.G.); kimheekyeong5378@gmail.com (H.-K.K.); naewoon159@naver.com (N.-U.K.); kimeunchae1008@naver.com (E.C.K.); dbgpwls01@naver.com (H.J.U.); 2 Division of Mechanical Engineering, College of Engineering, Wonkwang University, 460 Iksandae-ro, Iksan 54538, Jeonbuk, Republic of Korea; 3 MECHABIO Group, Wonkwang University, 460 Iksandae-ro, Iksan 54538, Jeonbuk, Republic of Korea

**Keywords:** polycaprolactone, calcium phosphate composite, nanostructure, mechano-bactericidal effect, cell behavior

## Abstract

Bone tissue engineering aims to develop biomaterials that are capable of effectively repairing and regenerating damaged bone tissue. Among the various polymers used in this field, polycaprolactone (PCL) is one of the most widely utilized. As a biocompatible polymer, PCL is easy to fabricate, cost-effective, and offers consistent quality control, making it a popular choice for biomedical applications. However, PCL lacks inherent antibacterial properties, making it susceptible to bacterial adhesion and biofilm formation, which can lead to implant failure. To address this issue, this study aims to enhance the antibacterial properties of PCL by incorporating calcium phosphate composite (PCL_CaP) nanostructures onto its surface via hydrothermal synthesis. The resulting “PCL_CaP” nanostructured surfaces exhibited improved wettability and demonstrated mechano-bactericidal potential against *Escherichia coli* and *Bacillus subtilis*. The flake-like morphology of the fabricated CaP nanostructures effectively disrupted bacteria membranes, inhibiting bacterial growth. Furthermore, the “PCL_CaP” surfaces supported the adhesion, proliferation, and differentiation of pre-osteoblasts, indicating their potential for bone tissue engineering applications. This study demonstrates the promise of calcium phosphate composite nanostructures as an effective antibacterial coating for implants and medical devices, with further research required to evaluate their long-term stability and in vivo performance.

## 1. Introduction

Bone tissue engineering is a promising field that is focused on developing biological substitutes to repair/regenerate diseased or damaged bone tissue [1]. One of the significant challenges in bone regeneration is designing scaffold materials that can replicate the structure, mechanical properties, and biological functions of natural bone while being biocompatible and osteoconductive to integrate with the tissue of the patient [2,3,4]. Surfaces using a combination of polymers and calcium phosphate (CaP) have been examined in order to develop surfaces with these functions [5,6,7,8]. Polycaprolactone (PCL), a biocompatible polymer, has emerged as one of the most widely used materials in tissue engineering and regenerative medicine, accounting for approximately 29% of scaffold materials due to its favorable properties, such as biocompatibility and biodegradability [9,10]. In addition, PCL has a relatively low melting point, making it compatible with several manufacturing techniques, such as electrospinning and 3D printing, to create scaffolds that mimic the complex structure of the native tissues [9,10,11,12,13]. Moreover, Food and Drug Administration (FDA) approval for biomedical applications further emphasizes its safety and long-term viability [2,14]. In addition, CaP has been reported to be osteoconductive and osteoinductive [5,6,7,8]. Furthermore, when mixed with biocompatible polymers, CaP can improve the mechanical properties of materials [15].

Despite these advantages, PCL and CaP materials lack inherent antibacterial properties, making them susceptible to biofilm formation [16]. Bacterial colonization on implant surfaces can occur within a short period post-implantation, leading to chronic infections that can impair scaffold function and, in severe cases, result in implant failure [17]. The formation of biofilms enhances bacterial resistance to antibiotics and the patient’s immune system, complicating infection management. Therefore, there has been a growing interest in developing biomaterials with intrinsic antibacterial properties for implantable devices [16,18,19,20].

To address this issue, various strategies have been explored to impart antibacterial functions to materials. These methods include incorporating antimicrobial agents, such as antibiotics or metal ions, into the material or using nanoparticles to prevent bacterial colonization [21,22,23,24,25]. However, this approach may increase bacterial resistance to antibiotics and potentially result in cytotoxicity to metal ions and nanoparticles [26]. Recent studies have shown that bacteria can be mechanically killed on nanostructured surfaces, such as the nanoscale pillar structures observed on cicada wings [27]. These mechanical antibacterial strategies physically disrupt bacterial membranes, preventing the emergence of resistant strains. This highlights the potential of nanostructures for mechano-bactericidal effects as a novel antibacterial strategy.

After the report of the mechanical bactericidal effect through nanostructures, research on the application of CaP nanorods to antibacterial implant surfaces has been reported. Nanorod-shaped CaP was grown on surfaces via hydrothermal synthesis and exhibited high antimicrobial properties against *P. aeruginosa* and low antimicrobial properties against *S. aureus* [28,29]. However, hydrothermal synthesis requires high pressure and heat to grow CaP nanostructures. Consequently, this approach is difficult to implement on polymer surfaces due to the high temperatures required. Therefore, studies on mechanical antimicrobial surfaces utilizing biocompatible polymers and CaP are still limited.

In this study, CaP composites, which are key components of bones and teeth, were synthesized in the form of nanostructures on the surface of the PCL polymer through a low-temperature and low-pressure hydrothermal synthesis using simulated body fluid (SBF) solution, and their effects on antibacterial properties and cell behavior were evaluated. CaP composites are widely known for their biocompatibility and osteoconductivity, and they are commonly used materials in bone-related applications [30,31]. They also have the advantage of promoting cell activity [3]. Our study aimed to introduce antibacterial properties to the polymer surface used in tissue engineering by utilizing the mechano-bactericidal effect of CaP nanostructures.

## 2. Materials and Methods

### 2.1. Materials

Polycaprolactone (PCL) (Mw: 45,000) was purchased from Sigma-Aldrich (St. Louis, MO, USA). The CaP composite nanostructures were synthesized through an SBF solution. The chemical reagents used in the preparation of the SBF solution are sodium chloride (NaCl), sodium hydrogen carbonate (NaHCO_3_), potassium chloride (KCl), di-potassium hydrogen phosphate trihydrate (K_2_HPO_4_·3H_2_O), magnesium chloride hexahydrate (MgCl_2_·6H_2_O), calcium chloride (CaCl_2_), sodium sulfate (Na_2_SO_4_), Tris (hydroxymethyl) aminomethane, and 1M hydrochloric acid solution (HCl). All the chemicals were purchased from Sigma Aldrich (St. Louis, MO, USA).

### 2.2. Prepartion of SBF Solution

The SBF solution was prepared according to a previously reported study [32]. In addition, to accelerate the biomimetic mineralization, the ionic concentration was increased by 5 times. The amounts of chemical reagents and the order of addition for 1 L of SBF solution are listed in Table 1. The SBF solution was prepared by dissolving the chemicals one by one in 700 mL deionized water (D.I. water), according to the sequence described in Table 1, while maintaining the temperature of the water at 37 °C. The pH of the solution was adjusted to 7.45 using Tris and 1M HCl. It is essential to maintain the pH within the range of 7.45 ± 0.3 throughout the preparation process.

### 2.3. Hydrothermal Synthesis of CaP Nanostructures

The “PCL bare” sample used as a control was fabricated using polished silicon (Si) wafers. First, PCL was melted at 90 °C and placed between two Si wafers, and the wafers were compressed to form a uniform PCL film. After cooling the film to room temperature, it was cut into squares measuring 10 × 10 mm^2^.

Prior to synthesizing CaP composite nanostructures on the PCL samples, they were pre-treated with a 1M NaOH solution for 30 min to induce surface activation and enhance wettability. This alkaline treatment is known to hydrolyze ester bonds in PCL, leading to the generation of OH/COOH functional groups on the surface, which promote nucleation and subsequent mineralization [33]. After the NaOH treatment, the samples were thoroughly rinsed with D.I. water and dried. The dried PCL samples were then immersed in SBF solution and maintained under continuous shaking at 100 rpm at 37 °C for 48 h to induce the formation of CaP composite nanostructures. The resulting surface was named as “PCL_CaP”.

### 2.4. Comprehensive Characterization of the “PCL_CaP” Nanostructured Surface: Structure, Chemical Composition, and Wettability

A multimodal analysis approach was employed to thoroughly characterize the surface of the “PCL_CaP” samples. These analyses were conducted to understand the structural, chemical, and surface wettability properties that influence the material’s biological performance and antibacterial properties.

#### 2.4.1. Morphological Analysis

The surface morphologies of the “PCL_CaP” samples were observed using scanning electron microscopy (SEM; S-4800, Hitachi, Tokyo, Japan). SEM was chosen due to its high resolution, allowing for a detailed visualization of the CaP nanostructures formed on the PCL surface. All samples were coated with a 5 nm layer of platinum using a sputtering coater (G20, GSEM, Suwon, Republic of Korea) prior to SEM imaging. The SEM images provided insight into the surface roughness and the distribution of CaP nanostructures, which are critical factors in promoting both osteointegration and antibacterial properties. The roughness average (Ra) of the synthesized CaP composite nanostructures was measured using Gwyddion 2.53 software. Gwyddion software is a professional software that is generally used to statistically evaluate the surface characteristics of SEM images [34]. The Ra value of the CaP composite nanostructures was measured using the magnified SEM image of the CaP composite nanostructures formed on the PCL surface. In addition, to observe the distribution of growth and the elemental composition of the CaP composite nanostructures, energy-dispersive spectroscopy (EDS) analyses were carried out.

#### 2.4.2. Chemical Bonding Analysis

Fourier transform infrared (FT-IR) spectroscopy was performed to identify the chemical composition of the “PCL_CaP” samples. FT-IR spectra were collected using single-reflection attenuated total reflection (ATR) mode on an FT/IR-4X spectrometer (Jasco, Tokyo, Japan) across a wavenumber range of 3500–500 cm^−1^ with a resolution of 4 cm^−1^. This method was selected to confirm the successful synthesis of CaP composite on the PCL surface by identifying characteristic vibrational bands of CaP composite. Additionally, the ATR mode allowed for precise surface-sensitive measurements, making it ideal for analyzing thin surface coatings and nanostructures.

#### 2.4.3. Surface Wettability Analysis

The wettability of the “PCL_CaP” surface was assessed by measuring the water contact angle (WCA) using a water droplet analyzer (SmartDrop_Plus, FemtoFAB, Seongnam, Republic of Korea). A 5 μL water droplet was placed on the surface at room temperature (23 °C, relative humidity 60%), and the contact angle was measured at five different locations on each sample to ensure accuracy and reproducibility. The WCA is an essential parameter for assessing surface hydrophilicity, which affects cell adhesion and proliferation.

### 2.5. Antibacterial Test of “PCL_CaP” Nanostructured Surfaces

The antibacterial properties of the “PCL_CaP” samples were evaluated using Gram-negative *Escherichia coli* (*E. coli*, ATCC 25404) and Gram-positive *Bacillus subtilis* (*B. subtilis*, ATCC 21332). Since mechanical sterilization via nanostructures mechanically damages the bacterial cell membrane, *E. coli* and *B. subtilis* bacteria were selected because they have a similar bacterial shape, motility, and different bacterial cell membrane thicknesses.

First, bacterial stocks were streaked onto Luria–Bertani (LB) agar plates and incubated for 18 h at 37 °C to obtain single colonies. A single colony was picked and inoculated from these plates into 10 mL of LB broth, followed by incubation in a shaking incubator set at 37 °C and 170 rpm. The bacterial suspension was diluted to an optical density (OD) of 0.3 at 600 nm (OD_600_ = 0.3) using LB medium. The sterilized samples were exposed to bacterial suspension for 5 h at 37 °C to allow bacterial attachment. After incubation, the samples were analyzed for antibacterial activity using live/dead staining, colony-forming unit (CFU) counting, and SEM imaging [35].

#### 2.5.1. Live/Dead Staining for Bacterial Viability

To assess bacterial viability, the samples cultured for 5 h in the bacteria suspension (OD_600_ = 0.3) were gently rinsed with sterile phosphate-buffered saline (PBS) to remove non-adherent cells. Then, a live/dead staining assay was performed using the BacLight™ Bacterial Viability Kit (L7012, Invitrogen, Waltham, MA, USA), which contains SYTO9 (for live bacteria) and propidium iodide (for dead bacteria). The samples were stained in the dark for 15 min and were subsequently mounted on glass slides for imaging. Bacterial viability and attachment were visualized using a confocal laser scanning microscope (LSM 980; Carl Zeiss, Oberkochen, Germany), and the results were quantified using ImageJ 1.53t software (NIH, Bethesda, MD, USA). This analysis provided insight into the ratio of live bacteria to dead bacteria on the surface of the “PCL_CaP” samples. The SEM and confocal images were analyzed by a core facility supporting the analysis and imaging of biomedical materials at Wonkwang University, supported by national research facilities and equipment centers.

#### 2.5.2. Colony-Forming Unit (CFU) Counting

CFU analysis was performed to quantify the survival of bacteria attached to the sample surface. Samples cultured in the bacterial suspension (OD_600_ = 0.3) for 5 h were collected and gently washed with sterile PBS. Then, they were carefully transferred to a conical tube containing 1 mL of sterile PBS. Ultrasonication (40 kHz, NXP-1002, Kodo, Hwaseong, Republic of Korea) was applied for 3 min to remove the bacteria attached to the sample surface. The bacterial suspensions were then serially diluted in PBS, and 100 µL of each dilution was evenly spread onto LB agar plates. These plates were incubated at 37 °C for 18 h, after which the number of bacterial colonies was counted to quantify bacterial viability on the sample surfaces. This method allowed for the precise determination of the antibacterial effect by comparing CFU counts from the “PCL_CaP” and control samples.

#### 2.5.3. SEM Imaging for Bacterial Morphology

For the morphological observation of attached bacteria, SEM was used. The samples were rinsed with PBS and then fixed in 4% paraformaldehyde (dissolved in PBS) for 15 min at 4 °C. After fixation, the samples underwent a dehydration process using a graded ethanol series (20%, 40%, 60%, 80%, and 100%) for 15 min at each concentration. This step ensured that the bacterial and sample structures were preserved without distortion during imaging. The fully dehydrated samples were then coated with a thin (5 nm) layer of platinum using a sputter coater to enhance conductivity and contrast for SEM observation.

### 2.6. Cell Viability Test

#### 2.6.1. Pre-Osteoblast Culture and Differentiation

MC3T3-E1 subclone 4 (CRL-2593) cells were obtained from the American Type Culture Collection (ATCC). For proliferation, the cells were maintained in Alpha-MEM (⍺-MEM) medium supplemented with 10% fetal bovine serum (FBS; Sigma-Aldrich, St. Louis, MO, USA) and 1% Antibiotic–Antimycotic solution (Sigma-Aldrich, St. Louis, MO, USA) at 37 °C with 5% (*v*/*v*) CO_2_. The culture medium was refreshed every two days. For osteogenic differentiation, the medium was further supplemented with L-ascorbic acid (50 µg/mL, Sigma-Aldrich, St. Louis, MO, USA) and β-glycerophosphate (10 mM, Sigma-Aldrich, St. Louis, MO, USA). The differentiation medium was also replaced every two days [36].

#### 2.6.2. Pre-Osteoblast Proliferation

MC3T3-E1 cells were seeded onto the prepared samples in 24-well plates at a density of 5 × 10^5^ cells per well in growth medium. The plates were incubated at 37 °C with 5% CO₂ for 3 and 7 days, with the medium being refreshed every two days. Cell viability was assessed using the Cell Counting Kit-8 (CCK-8, CK 04-20; Dojindo, Kumamoto, Japan). For the assay, 1 mL of 10% CCK-8 solution was added to each well and incubated at 37 °C with 5% CO_2_. OD_450_ was measured every 30 min for 4 h using a microplate reader (Infinite 200 PRO, TECAN, Maennedorf, Switzerland).

#### 2.6.3. Alkaline Phosphatase (ALP) Activity

To assess differentiation onto the prepared samples, an ALP activity analysis was conducted. MC3T3-E1 cells were seeded onto “PCL bare” and “PCL_CaP” samples in a 24-well plate at a density of 5 × 10^5^ cells per well in growth medium. After 24 h, the growth medium was replaced with a differentiation medium. The plates were incubated at 37 °C with 5% CO_2_ for predetermined intervals of up to 7 days. At the end of each time point, differentiation was evaluated using the TRACP & ALP Assay Kit (MK301; Takara, Kusatsu, Japan). Differentiated osteoblast cells were washed once with PBS, and 200 µL of extraction solution and substrate solution were added to each well. The samples were incubated at 37 °C for 45 min to allow for color development. Following incubation, 200 µL of stop solution was added to each well, and the absorbance was measured at 405 nm using a microplate reader. ALP activity was calculated based on the manufacturer’s instructions.

### 2.7. Statistical Analysis

All experimental data were collected in triplicate and are presented as mean ± standard deviation (SD). A one-way ANOVA test was used to compare three or more groups (*n* = 9), with statistical significance being established at *p* < 0.05.

## 3. Results and Discussion

### 3.1. Characterization of “PCL_CaP” Nanostructured Surfaces

**Figure 1:**
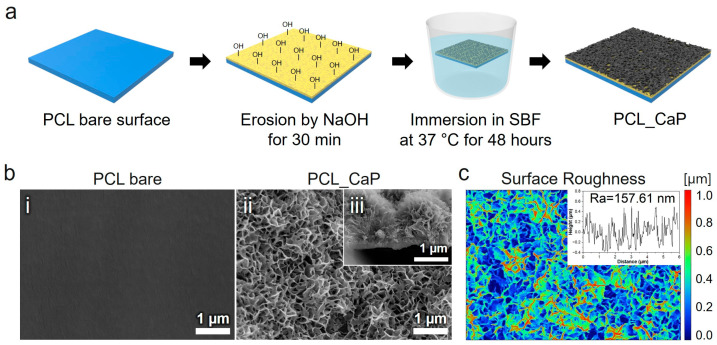
(**a**) Schematic diagram of the preparation of “PCL_CaP” nanostructured surfaces. (**b**) Representative SEM images showing the (**i**) surface of “PCL bare”, (**ii**) surface of “PCL_CaP”, and (**iii**) cross section of “PCL_CaP.” (**c**) Surface roughness for the “PCL_CaP” nanostructured surface.

The detailed process of fabricating the “PCL_CaP” nanostructured surfaces is illustrated in Figure 1a. This schematic diagram outlines each step involved in the preparation, providing a clear understanding of the methodology employed in this study. Initially, the PCL surface was treated with NaOH to induce surface hydrolysis to increase surface roughness and generate hydroxyl (OH^−^) groups [33]. These OH groups are crucial as they create reactive sites that facilitate the nucleation of CaP in the subsequent steps. Without these OH groups, the formation of CaP on the PCL surface would be inefficient due to the lack of reactive sites for calcium and phosphate ions to attach [32]. After the NaOH treatment, the PCL surface was immersed in an SBF solution. This solution is ionically similar to human blood plasma, containing calcium (Ca^2^⁺) and phosphate (PO_4_^3^⁻) ions in concentrations that promote biomineralization. The reaction that occurs in the SBF solution involves the adsorption of these ions onto the hydroxylated PCL surface, followed by the precipitation of CaP nanostructures. The formation of CaP nanostructures is driven by the supersaturation of calcium and phosphate ions in the SBF, which results in the nucleation and growth of CaP crystals [2,32]. This process mimics the natural biomineralization that occurs in bone tissue, making it highly relevant for biomedical applications [1].

The surface morphology of the synthesized CaP nanostructures on the PCL surface was meticulously examined using SEM. Figure 1b(i) show a clean and uniformly flat surface, which was used as the control sample. As depicted in Figure 1b(ii), the SEM images demonstrate a uniform distribution of nanostructures across the PCL surface. The observed CaP nanostructures exhibit a well-defined flake-like shape [2,37], which is indicative of successful synthesis via the hydrothermal method (Figure 1b(iii)). This uniformity and distinct crystalline structure suggest that the hydrothermal conditions were optimal for CaP formation, contributing to the potential biological efficacy of the modified PCL surface. The surface roughness of the “PCL_CaP” samples was measured using Gwyddion 2.53 software with average values of Ra = 157.61 nm (Figure 1c).

**Figure 2:**
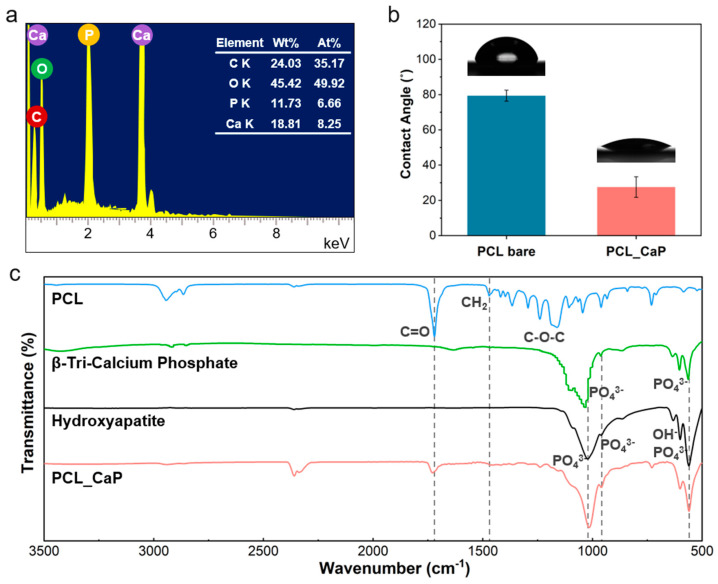
(**a**) EDS images; (**b**) WCA of the “PCL_CaP” nanostructured surface; (**c**) FT-IR spectra (including PCL, β-Tri-Calcium Phosphate, hydroxyapatite, and “PCL_CaP”).

To further understand the elemental composition of the “PCL_CaP” surface, an EDS analysis was conducted. The EDS results in Figure 2a confirmed the presence of critical elements associated with CaP calcium (Ca), phosphorus (P), and oxygen (O). These findings align with the expected composition of CaP, reinforcing the success of the synthesis process [38]. The quantitative analysis revealed the following elemental weight percentages: 24.03% carbon (C), 45.42% oxygen (O), 11.73% phosphorus (P), and 18.81% calcium (Ca). These results not only validate the presence of CaP, however, also highlight the potential of the modified PCL surface for applications in bone tissue engineering.

The hydrophilicity of the “PCL_CaP” surface was evaluated by measuring the WCA, which is a critical parameter for assessing the material’s suitability for biomedical applications [39]. The measurements, illustrated in Figure 2b, demonstrate a significant reduction in the WCA of the “PCL_CaP” surface when compared to the control sample “PCL bare”. Specifically, the contact angle value for “PCL bare” was recorded at 79.36°, whereas the “PCL_CaP” surface exhibited a markedly lower contact angle of 27.58°. This substantial decrease in WCA can be attributed to two main factors, the intrinsic properties of CaP and surface morphology. CaP is a naturally hydrophilic material, and its incorporation onto the PCL surface increases the surface’s overall hydrophilicity by providing polar sites that can readily interact with water molecules [40]. Additionally, the presence of nanostructures significantly increases surface roughness at the nanoscale. This roughness leads to a larger surface area for water to contact and spread across, further reducing the contact angle [41]. These combined effects not only improve the wettability of the “PCL_CaP” surface but also enhance its ability to support biological functions, such as cell adhesion and proliferation, making the material more suitable for tissue engineering applications [3].

FT-IR spectroscopy was conducted to verify the formation of CaP on the PCL surface. The spectra of PCL, natural hydroxyapatite (HA) powder, β-Tri-Calcium Phosphate (β-TCP), and “PCL_CaP” are presented in Figure 2c. HA and β-TCP belong to the group of calcium phosphate compounds. Therefore, HA and β-TCP were additionally measured to compare whether the calcium phosphate compound synthesized using an SBF solution of five times falls within the category of calcium phosphate compound.

The PCL spectrum exhibits characteristic peaks at 1720 cm^−1^ (C=O stretching), 1460 cm^−1^ (CH_2_ bending), and 1100–1300 cm^−1^ (C–O–C stretching), corresponding to the typical structure of PCL [42]. In the spectrum of natural HA powder, distinct peaks at 960 cm^−1^ and 1030–1100 cm^−1^ are observed, attributed to the symmetric and asymmetric stretching of phosphate (PO_4_^3−^) groups, along with a peak at 630 cm^−1^, corresponding to the hydroxyl (OH^−^) group [4]. Similarly, the β-TCP spectrum exhibits comparable PO_4_^3−^ peaks; however, slight variations in intensity and position distinguish it from HA, reflecting differences in their crystalline structures [43]. The “PCL_CaP” spectrum reveals additional peaks at 960 cm^−1^, 1030–1100 cm^−1^ (PO_4_^3−^ groups), and 630 cm^−1^ (OH^−^ group), aligning closely with those found in β-TCP. These peaks confirm the successful mineralization of CaP on the PCL surface. Furthermore, the retention of key PCL peaks (C=O, CH_2_, and C–O–C) in the “PCL_CaP” spectrum indicates that SBF treatment facilitated CaP formation without compromising the structural integrity of PCL.

### 3.2. Evaluation of the Antibacterial Properties of “PCL_CaP” Nanostructured Surfaces

**Figure 3:**
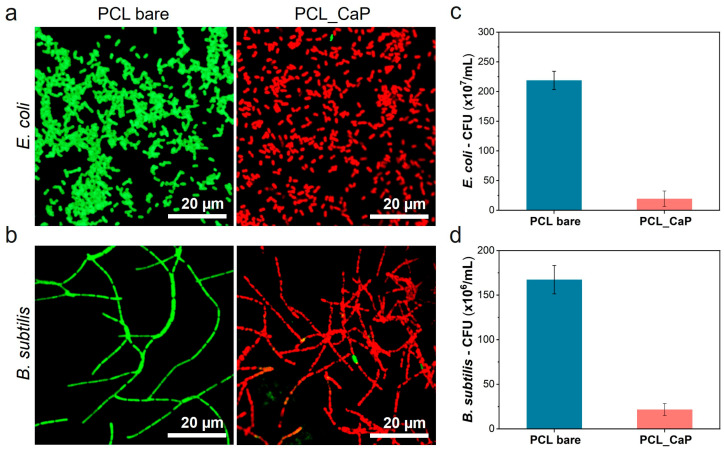
Confocal microscopy images of (**a**) *E. coli* and (**b**) *B. subtilis* cultured on the “PCL bare” surface and the “PCL_CaP” nanostructured surface (green: live cells; red: dead cells). CFU of (**c**) *E. coli* and (**d**) *B. subtilis* cultured on the “PCL bare” surface and the “PCL_CaP” nanostructured surface.

Figure 3 shows the results of the antibacterial properties of the “PCL_CaP” nanostructured surface. Confocal microscopy was used to visualize the viability of bacteria on the “PCL bare” and “PCL_CaP” surfaces. As shown in Figure 3a,b, green fluorescence corresponds to live cells, while red fluorescence indicates dead cells. Notably, the confocal images reveal a significantly higher proportion of dead bacteria on the “PCL_CaP” surfaces compared to the “PCL bare” surfaces. This observation suggests a pronounced antibacterial effect of the CaP nanostructures, leading to increased cell death upon contact.

The antibacterial activity was quantitatively confirmed through CFU assays, as shown in Figure 3c,d. Both *E. coli* and *B. subtilis* exhibited a substantial reduction in colony formation on the “PCL_CaP” surfaces compared to the control. Specifically, the bacterial count on the “PCL_CaP” surface was (19.4 ± 13.0) × 10^7^ CFU/mL, corresponding to an antibacterial efficacy of 91.1% compared to the count of (218.8 ± 15.3) × 10^7^ CFU/mL observed on the “PCL bare” surface against *E. coli*, as shown in Figure 3c. For *B. subtilis*, the antibacterial rate was 87.0%, with a bacterial count of (21.7 ± 6.7) × 10^6^ CFU/mL on the “PCL_CaP” surface compared to (167.3 ± 15.9) × 10^6^ CFU/mL on the “PCL bare” surface in Figure 3d. The bactericidal activity of CaP nanostructures against *B. subtilis* was lower than that against *E. coli* bacteria. This result is thought to be because *B. subtilis*, a Gram-positive bacterium, has a thick peptidoglycan layer [44]. Mechanical bactericides have a mechanism for sterilizing by directly deforming the bacteria membrane attached to the nanostructured surface [45]. However, if the membrane of the bacteria is thick, the deformation caused by the nanostructures may not be significant, which may decrease the bactericidal activity.

**Figure 4:**
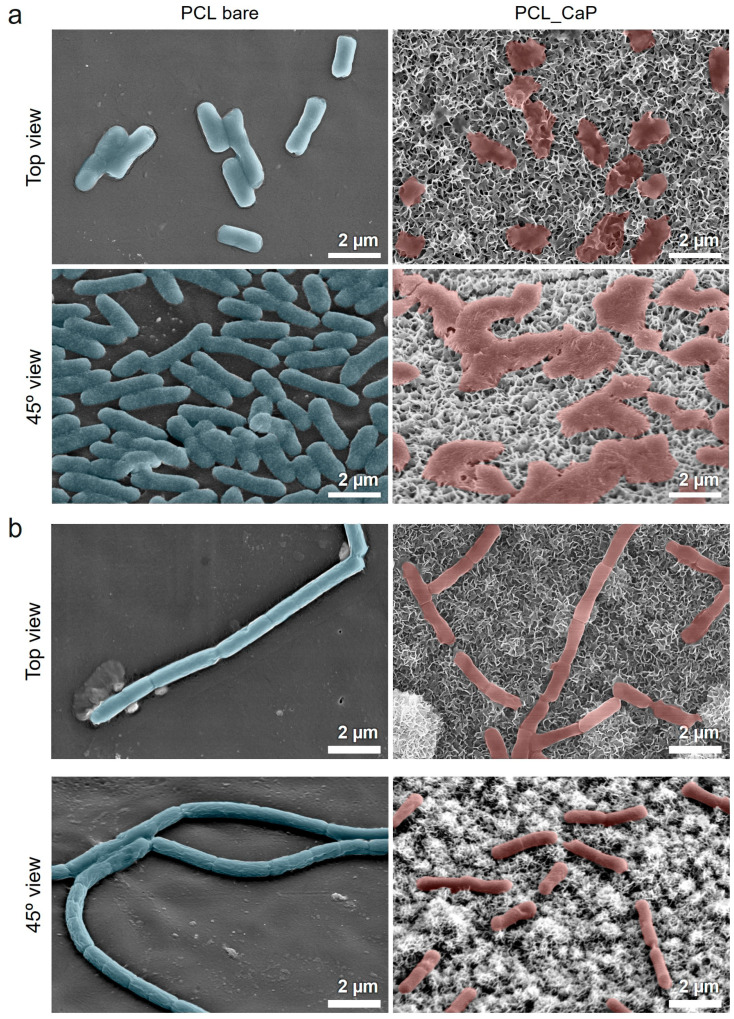
SEM images of (**a**) *E. coli* and (**b**) *B. subtilis* cultured on the “PCL bare” surface and “PCL_CaP” nanostructured surface.

The bacterial mechanical bactericidal effect has a mechanism in which the bacterial membrane attached to the nanostructure surface is penetrated or stretched by the nanostructure [46]. Therefore, we used the SEM image to confirm the membrane morphology of the bacteria attached to the “PCL_CaP” nanostructure surface. Figure 4a shows SEM images of *E. coli* cultured on the “PCL bare” and “PCL_CaP” surfaces. On the “PCL bare” surface, it seems that the membranes are not damaged at all, and the bacteria maintain their characteristic rod-shaped morphology. On the “PCL_CaP” surface, it seems that *E. coli* sank due to membrane damage induced by the nanostructures. This shows that the sharp flake-like CaP nanostructures have a strong bactericidal effect, and the “PCL_CaP” surface has a sufficient mechanical stiffness to damage the attached bacterial membrane. As a Gram-negative bacterium, *E. coli* has a relatively thin membrane composed of a lipopolysaccharide (LPS) layer [47], which provides limited resistance against the mechanical forces exerted by sharp nanostructures. This structural vulnerability results in a higher degree of deformation and rupture.

Figure 4b shows SEM images of *B. subtilis* cultured on the “PCL bare” and “PCL_CaP” surfaces. On the “PCL bare” surface, *B. subtilis* maintained its smooth, rod-shaped morphology, indicating that the surface induces little to no stress on the bacteria membrane. In contrast, the “PCL_CaP” surface shows minor deformation and surface irregularities, but the overall shape of *B. subtilis* remains largely intact. Unlike *E. coli*, *B. subtilis* is a Gram-positive bacterium with a thick peptidoglycan layer that serves as a mechanical barrier against external forces. The rigidity and thickness of this peptidoglycan layer reduce the extent of deformation caused by the CaP nanostructures. As a result, the mechanical stress exerted by the nanostructures is less effective at penetrating and rupturing the bacterial membrane. These structural differences explain why CaP nanostructures exhibit relatively low bactericidal efficacy against *B. subtilis*.

### 3.3. Pre-Osteoblast Behavior of “PCL_CaP” Nanostructured Surfaces

**Figure 5:**
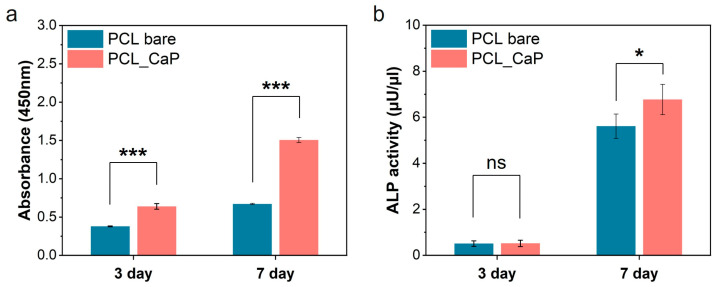
Assessment of pre-osteoblast proliferation and differentiation. (**a**) CCK-8 assay (proliferation); (**b**) ALP activity (differentiation). (ns; not significant, *; *p* < 0.05, ***; *p* < 0.001).

Calcium ions released from the calcium phosphate surface are reported to be related to cell proliferation and differentiation, which can affect the nutrient supply to cells and cell chemotaxis [48]. To assess pre-osteoblast proliferation and differentiation in relation to CaP nanostructures, CCK-8 assays and ALP activity analyses were conducted. To evaluate osteoblast proliferation in relation to the “PCL_CaP” nanostructures, MC3T3-E1 cells were cultured for 7 days, and a CCK-8 assay was performed (Figure 5a). The proliferation of pre-osteoblasts on all surfaces showed a steady increase over 3 and 7 days. Notably, osteoblast proliferation on the “PCL_CaP” nanostructured surface showed a significant increase at both 3 and 7 days. Topological features and surface roughness at the micrometer and nanometer scales affect cell behavior, as these dimensions align with the sizes of individual cells, integrin clusters, and proteins [49]. Additionally, the increased surface area on rougher surfaces led to the greater adsorption of serum proteins, which may enhance cell attachment and promote proliferation across a larger area [50]. The “PCL_CaP” nanostructured surface exhibits a greater surface roughness and an increased surface area compared to “PCL bare”, owing to the presence of CaP nanostructures. As previously noted, the enhanced surface area facilitates improved cell attachment and proliferation. Consequently, it was demonstrated that the “PCL_CaP” nanostructured surface significantly enhances pre-osteoblast attachment and proliferation compared to “PCL bare”.

Moreover, ALP activity analysis was performed to assess pre-osteoblast differentiation in relation to the “PCL_CaP” nanostructured surface. ALP activity is strongly linked to the mineralization capacity of osteoblasts, serving as a valuable marker for assessing pre-osteoblast differentiation [51]. As shown in Figure 5b, on day 3, there was no significant difference in ALP activity between the “PCL bare” and the “PCL_CaP” nanostructured surface. However, by day 7, ALP activity was noticeably higher in the “PCL_CaP” nanostructured surface. As mentioned above, CaP is well recognized for its biocompatibility and osteoconductivity, making it a widely used material in bone-related applications. Additionally, it has the benefit of enhancing osteoblast activity [52,53,54]. Therefore, it was confirmed that the “PCL_CaP” nanostructured surface enhances osteoblast differentiation through its osteoconductive properties. Consequently, the “PCL_CaP” nanostructured surface not only promotes pre-osteoblast attachment and proliferation by increasing surface area but also supports osteoblast differentiation through its osteoconductivity.

## 4. Conclusions

In this study, we propose an antibacterial surface through low-temperature hydrothermal synthesis using biocompatible polymers PCL and calcium phosphate. The resulting “PCL_CaP” nanostructured surfaces exhibited distinct morphological, chemical, and physical characteristics, as confirmed through various analytical techniques, including SEM, EDS, and FT-IR. Notably, the CaP nanostructures significantly enhanced the wettability of the PCL surfaces, suggesting improved potential for biological applications. The fabricated “PCL_CaP” surface exhibits effective antibacterial activity against *E. coli* and *B. subtilis*. This activity is attributed to the disruption of bacterial membranes, ultimately leading to bacteria death, facilitated by the flake-like morphology of the CaP nanostructures. Additionally, the “PCL_CaP” nanostructured surface not only promoted pre-osteoblast adhesion and proliferation, but also supported pre-osteoblast differentiation. This study highlights the potential of CaP nanostructures as a promising antibacterial agent for biomedical applications, particularly in the development of antibacterial coatings for implants and medical devices. Future work will focus on assessing the long-term stability of the CaP nanostructured surfaces and evaluating their performance in in vivo models to further establish their efficacy and safety for clinical applications.

## Figures and Tables

**Table 1:** Recipe for 1 L of SBF solution used in this study.

**Order**	**Reagent**	**Amount**
1	NaCl	40.175 g
2	NaHCO_3_	1.775 g
3	KCl	1.125 g
4	K2HPO_4_·3H_2_O	1.155 g
5	MgCl_2_·6H_2_O	1.555 g
6	1M HCl	195 mL
7	CaCl_2_	1.46 g
8	Na_2_SO_4_	0.36 g
9	Tris	30.59 g
10	1M HCl	0~25 mL

## Data Availability

Data are contained within the article.
